# Clinical-Pathological Conference Series from the Medical University of Graz: Case No 175: A 54-year-old man with hyponatremia and delirium after surgery for a prolapsed disc

**DOI:** 10.1007/s00508-022-02097-w

**Published:** 2022-11-08

**Authors:** Elisabeth Fabian, Anja Reisinger, Werner Ribitsch, Vanessa Stadlbauer, Andreas J. Eherer, Regina Roller-Wirnsberger, Hermann Toplak, Peter Fickert, Guenter J. Krejs

**Affiliations:** 1 Department of Internal Medicine II, University Hospital Krems, Karl Landsteiner University of Health Sciences, Krems on the Danube, Austria; 2 Department of Internal Medicine, Hartberg State Hospital, Hartberg, Austria; 3 Division of Nephrology, Department of Internal Medicine, Medical University of Graz, Graz, Austria; 4 Division of Gastroenterology and Hepatology, Department of Internal Medicine, Medical University of Graz, Auenbruggerplatz 15, 8036 Graz, Austria; 5 Department of Internal Medicine, Medical University of Graz, Graz, Austria; 6 Division of Endocrinology and Diabetology, Department of Internal Medicine, Medical University of Graz, Graz, Austria

**Keywords:** Acute intermittent porphyria, SIADH, Hemin infusion, Givosiran, Constipation

## Presentation of case

**Dr. A. Reisinger:** The patient complained of sciatica sensations affecting the dermatomes of L4/5 bilaterally and partly of S1 on the right leg. Magnetic resonance imaging (MRI) of the spine showed a massive disc prolapse at the level of L3/4 on the right. Flavectomy and disc extraction were performed without complications at Graz University Medical Center. After surgery, the patient developed severe constipation for which he received laxatives and colonic lavage. Furthermore, blood work showed hyponatremia (123 mmol/L, normal: 135–145 mmol/L), which was thought to result from dilution effects. Since the postoperative course was otherwise unremarkable, the patient was discharged 5 days after surgery; however, 3 days later, the patient was admitted to a regional hospital because the recommended serum electrolyte measurement ordered by his family physician had shown marked hyponatremia (118 mmol/L). At that time, the patient was tired and not alert. His wife reported that he had been disoriented and confused in the morning prior to admission. Later that day, the patient developed acute delirium with visual hallucinations and was transferred to the intensive care unit for careful sodium replacement.

The patient is a musician. He drinks hardly any alcohol because it gives him a prolonged hangover with a headache and abdominal pain. His past medical history was unremarkable except for chronic constipation and a urinary tract infection with suspected macrohematuria and pain in the lower abdomen 6 months earlier.

On admission, the patient (body height: 186 cm, body weight: 86 kg, body mass index 25 kg/m^2^) was afebrile but in a reduced general health condition. His blood pressure was 151/81 mm Hg, heart rate 75 bpm and oxygen saturation 98% at ambient air. Physical examination revealed a sensorimotor deficit at the lateral aspect of the right thigh, at the lower leg and the first toe, but was otherwise unremarkable (including a cursory neurologic examination). His medication included metamizole for postoperative pain management, a proton pump inhibitor and simethicone soft tablets.

Laboratory: Leukocytes 8.3 × 10^9^/L (normal: 4.0–10.0 × 10^9^/L), erythrocytes 4.13 × 10^12^/L (normal: 4.50–5.90 × 10^12^/L), hemoglobin 13.0 g/dL (normal: 14.0–17.5 g/dL), hematocrit 31.9% (normal: 40.0–50.0%), mean corpuscular volume 77 fL (normal: 80–98 fL), mean corpuscular hemoglobin 31.5 pg (normal: 28.0–32.0 pg), platelets 155 × 10^9^/L (normal: 140–440 × 10^9^/L), unremarkable differential blood count, C‑reactive protein (CRP) 0.6 mg/L (normal: < 5.0 mg/L), sodium 114 mmol/L (normal: 135–145 mmol/L), potassium 3.35 mmol/L (normal: 3.50–5.00 mmol/L), chloride 73 mmol/L (normal: 95–110 mmol/L), total calcium 2.24 mmol/L (normal: 2.20–2.75 mmol/L), magnesium 0.51 mmol/L (normal: 0.70–1.05 mmol/L), creatinine 0.83 mg/dL (normal: 0.50–1.20 mg/dL), urea 33 mg/dL (normal: 10–50 mg/dL), uric acid 3.6 mg/dL (normal: 3.4–7.0 mg/dL), glomerular filtration rate 99 mL/min/1.73 m^2^ (normal: 80–140 mL/min/1.73 m^2^), total bilirubin 1.10 mg/dL (normal: < 1.20 mg/dL), alkaline phosphatase 74 U/L (normal: 40–129 U/L), gamma-glutamyl transferase (GGT) 75 U/L (normal: 10–71 U/L), cholinesterase 6451 U/L (normal: 4600–13,000 U/L), aspartate amino transferase (AST) 33 U/L (normal: 0–35 U/L), alanine amino transferase (ALT) 35 U/L (normal: 0–45 U/L), lactate dehydrogenase 329 U/L (normal: 135–225 U/L), glucose 103 mg/dL (normal: 60–110 mg/dL), prothrombin time 102% (normal: 70–140%), activated partial thromboplastin time (aPTT) 24 sec (normal: 25–37 sec), total protein 6.94 g/dL (6.60–8.30 g/dL), albumin 4.33 g/dL (3.50–5.20 g/dL), serum osmolality 241 mosmol/kg (normal: 280–300 mosmol/kg). Hormones: thyroid-stimulating hormone (TSH) 1.44 μU/mL (normal: 0.27–4.20 µU/mL), cortisol 223.1 ng/mL (normal: 43.0–220.0 ng/mL), aldosterone < 3.7 ng/dL (normal: 3.7–43.2 ng/dL), renin 3.7 μU/mL (normal: 5.3–99.1 µU/mL), adrenocorticotropic hormone (ACTH) 34.5 pg/mL (normal: 10.0–51.0 pg/mL), antidiuretic hormone 3.7 pg/mL (normal: 0.0–6.7 pg/mL). Urine: sodium 128 mmol/L, potassium 25 mmol/L, chloride 112 mmol/L, calcium 4.80 mmol/L, urine osmolality 471 mosmol/kg (normal: 50–1200 mosmol/kg), urinary test stick: bilirubin +++, urine sediment examination was without pathologic findings.

Electrocardiogram showed normal sinus rhythm. Computed tomography (CT) of the chest revealed a normal structure of the lung and the bronchial tree without pathologically enlarged lymph nodes. MRI of the brain was also without pathologic findings.

The consulted psychiatrist made the diagnosis of delirium with psychomotor symptoms and recommended treatment with risperidone, pregabalin and lorazepam. A flat plate of the abdomen showed considerable gas in the small bowel and colon and marked coprostasis.

A diagnostic test was performed.

## Differential diagnosis

**Dr. W. Ribitsch:** This is a challenging case of a musician who underwent flavectomy and disc extraction because of symptomatic disc prolapse. After surgery, he developed severe constipation for which he received laxatives and colonic lavage, and hyponatremia (114 mmol/L) which was initially thought to result from dilution effects. Clinical deterioration with fatigue, mental confusion and acute delirium with visual hallucinations finally led to admission to the intensive care unit for careful sodium replacement. His past medical history was unremarkable except for chronic constipation and a urinary tract infection with suspected macrohematuria and pain in the lower abdomen 6 months prior to admission. The patient drinks hardly any alcohol because it gives him a prolonged hangover with symptoms such as abdominal pain and headache. Laboratory data showed marked hyponatremia (114 mmol/L) but also slightly decreased serum levels of potassium and magnesium, and decreased serum osmolality (241 mosmol/kg). Serum glucose, renal function parameters, liver parameters and pituitary hormones were all within normal limits. Analysis of urine revealed elevated levels of sodium (128 mmol/L) and potassium, and increased urine osmolality (471 mosmol/kg).

Given that preoperative blood tests showed normal serum electrolytes, the development of hyponatremia within 48 hrs after surgery constitutes acute hyponatremia in this case. In contrast, chronic hyponatremia, which is more frequently seen in clinical practice than acute hyponatremia, evolves over a period longer than 48 hrs. Additional consideration of the reduced serum osmolality finally defines acute hypoosmolar hyponatremia in the presented case. In order to narrow down the differential diagnosis in patients with hypoosmolar hyponatremia, the volume status has to be assessed. In our patient it was normal. In hypervolemic patients, hypoosmolar hyponatremia may be due to heart failure, liver failure, acute renal failure or nephrotic syndrome. In hypovolemic patients with hypoosmolar hyponatremia, the underlying causes to consider include cerebral salt wasting, salt losing nephropathy, primary adrenal insufficiency, chronic kidney disease and intake of diuretics [1]. To differentiate reliably between hypovolemic and euvolemic hypoosmolar hyponatremia, urinary sodium excretion has to be assessed as well. Under physiological conditions, hypovolemia leads to increased release of antidiuretic hormone from the pituitary gland and activation of the renin-angiotensin-aldosterone system, which subsequently causes an increase of urine osmolality and a reduction of urinary excretion of sodium (< 30 mmol/L). In contrast, patients with euvolemic hypoosmolar hyponatremia show urinary sodium levels > 40 mmol/L [2]. Clinically, they present without edema and with a normal blood pressure. Hypoosmolar hyponatremia (serum osmolality < 280 mosmol/kg) and euvolemia suggests cortisol deficiency, hypothyroidism or syndrome of inappropriate antidiuretic hormone secretion (SIADH) as potential underlying causes of our patient’s condition [2]. Clinical features of cortisol deficiency include psychiatric symptoms (delirium) and gastrointestinal complaints such as nausea, vomiting, diarrhea and constipation that were also seen in the discussed patient; however, a negative history of glucocorticoid intake, a lack of clinical signs of an Addisonian crisis and normal blood levels of pituitary hormones make cortisol deficiency an unlikely diagnosis in this case. Hypothyroidism is associated with a wide range of clinical symptoms and implications. It affects all aspects of metabolism (e.g. weight gain, cold intolerance, fatigue) and the gastrointestinal tract (e.g. constipation), and may lead to neuropsychiatric symptoms and electrolyte disturbances (e.g. hyponatremia). In addition, hypothyroidism exerts unfavorable effects on the cardiovascular system but also on the neurosensory, musculoskeletal, endocrine and hematologic systems in the human body [3]. Patients with latent hypothyroidism are prone to hypothyroid crises, especially in stressful situations such as surgery, trauma or infections. Although some of the mentioned complications of hypothyroidism could be identified in the discussed patient, it should actually be excluded as a differential diagnosis, because in this condition hyponatremia occurs only rarely, is usually mild and does not cause an acute delirium with hallucinations as seen in this case. Finally, a normal serum level of TSH as found in the discussed patient de facto rules out hypothyroidism. This leaves SIADH as the most likely differential diagnosis for euvolemic hypoosmolar hyponatremia. Indeed, the patient fulfills all essential criteria defined for this diagnosis, i.e. effective serum osmolality < 275 mosmol/kg, urine osmolality > 100 mosmol/kg, euvolemia, urine sodium concentration > 30 mmol/L with normal dietary salt and water intake, absence of adrenal, thyroid, pituitary or renal insufficiency, and no intake of diuretics [1]; however, the findings of hypokalemia, hypomagnesemia, acute delirium and constipation in our patient do not fit this diagnosis. There are multiple causes of SIADH including various malignancies and infections, but also disorders of the nervous system such as multiple sclerosis and Guillain-Barré syndrome, acute intermittent porphyria and a wide range of drugs including antidepressants, anticonvulsants, antipsychotics and anticancer drugs. According to the literature, proton pump inhibitors are also among the drugs that may induce SIADH [1]. Although the discussed patient had taken such medication, it seems unlikely that this was the reason for his condition because he had been on the said treatment for a longer period of time without experiencing any adverse effects.

In summary, the constellation of symptoms of constipation, acute delirium and hyponatremia, along with the history of a prolonged hangover with abdominal pain and headache after drinking alcohol, suggests the diagnosis of acute intermittent porphyria [4]. Moreover, the patient presented with a “highly elevated bilirubin” in urine, but a normal serum bilirubin level, which may be a false positive result of the urinary test stick due to excretion of porphyrins. In addition, the history of suspected macrohematuria hints to an earlier episode of acute intermittent porphyria as urine of these patients can appear red or brown after exposure to oxygen, light or heat due to conversion of porphobilinogen into porphobilin [5, 6]. Porphyrias are clinical syndromes that arise due to deficiency or defect in a particular enzyme involved in a specific step of the heme synthesis pathway. The clinical presentation, severity and prognosis of individual porphyrias depend on which enzyme is deficient and the corresponding heme precursor or porphyrin accumulation [7]. Porphyrias can be classified as hepatic or erythropoietic depending on the primary location of overproduction or accumulation of the porphyrin precursors (delta-aminolevulinic acid, porphobilinogen) or porphyrin (uroporphyrin, coproporphyrin, protoporphyrin) [8]. Acute porphyrias are due to hepatic overproduction of the porphyrin precursors delta-aminolevulinic acid and porphobilinogen, and primarily manifest with systemic symptoms of neurovisceral pain (pseudoacute abdomen), neuropathy and mental disturbances. In contrast, erythropoietic porphyrias are characterized by cutaneous photosensitivity due to overproduction of photosensitizing porphyrins by the liver and bone marrow [5]. Two of these, variegate porphyria and hereditary coproporphyria, have both neurovisceral and cutaneous features [8]. Acute intermittent porphyria is the acute type most often encountered in clinical practice [9]. It results from heterozygous mutations in the hydroxymethylbilane synthase (HMBS) gene, leading to deficiency of porphobilinogen deaminase (PBGD, also known as hydroxymethylbilane synthase), which is an enzyme that plays an integral role in heme synthesis. Today, about 390 HMBS gene mutations are known [10]. Exposure to certain triggers such as alcohol, infections, caloric restriction, change in reproductive hormones or certain medication will amplify the activity of delta-aminolevulinic acid synthase as an attempt to increase heme synthesis. This leads to accumulation of delta-aminolevulinic acid and porphobilinogen, and causes acute attacks with the typical manifestations of pseudoacute abdomen, constipation, psychiatric disturbances and neuropathy [8]. The attacks have a recurring character. Hyponatremia is a common but nonspecific laboratory finding associated with the onset of an attack of acute intermittent porphyria. It has been documented in 25–60% of cases and is probably multifactorial (elevated levels of antidiuretic hormone and intestinal and/or renal sodium loss during a crisis) [11, 12].

In view of the entire constellation of findings in this case, acute intermittent porphyria seems to be the most likely diagnosis. This should be confirmed by the analysis of porphobilinogen and delta-aminolevulinic acid in urine. In addition, genetic testing should be performed to identify the specific mutation and form of porphyria.

## Dr. W. Ribitsch’s diagnosis

Acute intermittent porphyria

## Discussion of case

**Dr. V. Stadlbauer:** As summarized by Dr. Ribitsch, this case strongly suggests the diagnosis of acute intermittent porphyria. To confirm this, porphyrins and their precursors were analyzed in 24‑h urine (Table 1) and found to be elevated. In addition, genetic testing revealed a pathogenic heterozygous mutation of the HMBS gene (exon 9, C.580 C > TP, GLN 194*). After treatment with hemin (4 mg/kg body weight for 4 days), the patient’s symptoms disappeared; after an additional 3 months even the paresthesia vanished completely.

**Table 1:** Urinary porphyrins and porphyrin precursors in the discussed patient

Substance	Value	Normal range
Porphyrins (µg/24‑h urine)	576.3	0.0–150.0
Delta-aminolevulinic acid (µg/24‑h urine)	39.95	0.25–6.40
Porphobilinogen (mg/24‑h urine)	121.71	0.10–1.70

The management of a patient with acute intermittent porphyria typically starts with termination of potential causative medications and intravenous administration of glucose (300–400 g/24 h), which inhibits transcription of the gene encoding transhepatic 5’-aminolevulinate synthase 1 (ALAS1) [5, 9]. Parenteral opioids are sometimes used in managing abdominal pain. Intravenous administration of hemin (3–4 mg/kg body weight) is recommended without delay in severe acute attacks and should be maintained for 4 days [5, 13]. A response is usually seen on the third day with a decrease of urine and serum porphobilinogen [7]. Hemin (also hematin; chemically: ferric chloride heme, i.e. protoporphyrin IX containing a ferric iron (Fe^3+^) ion with a coordinating chloride ligand) replenishes the hepatocyte heme pool and downregulates ALAS1, which results in reduced production of porphyrin precursors and corresponding improvement in symptoms [14]. Besides downregulating hepatic ALAS1 transcription, administered hemin also exerts effects on ALAS1 by inducing mRNA destabilization and blocking mitochondrial import of the mature enzyme [15]. Despite disadvantages such as iron accumulation and the risk of thrombophlebitis, preventive therapy with regular hemin infusions is used to treat acute intermittent porphyria with recurrent attacks [16, 17].

Hemin was first synthesized by Hans Fischer (1881–1945), a German organic chemist and physician who was awarded the Nobel Prize in Chemistry in 1930 for his research on the molecular structure of hemin and chlorophyll. Fischer had shown as early as in 1915 that urine and feces of a case of congenital porphyria, a disease then recently discovered, contained uroporphyrin and coproporphyrin. He held the Chair of Medical Chemistry in Innsbruck, Austria (1916–1918) and Vienna, Austria (1918–1921); from 1921 until his death he was Professor of Organic Chemistry at the Technical College in Munich, Germany [18].

Recently, a novel treatment to prevent attacks of acute intermittent porphyria was developed. Givosiran is an RNA interference therapy targeting ALAS1 messenger RNA (thus, reducing the hepatic production of delta-aminolevulinic acid and porphobilinogen), which was approved in Europe in early 2020. In a randomized trial, givosiran significantly reduced the mean annual attack rate from 12.5 to 3.2 as compared with placebo [19]. This therapy is indicated in patients who present with two or more attacks within 6 months. In Austria, the first patient with acute intermittent porphyria was treated with givosiran at Graz University Medical Center in June of 2021. This 55-year-old woman was first diagnosed with acute intermittent porphyria in 1994. Because she experienced more than 30 acute attacks between 1994 and 1999, the patient was managed with a regimen of two doses hemin (250 mg) every other week. Due to the toxic effect of this substance on veins, the patient needed a total of 12 port-a-caths during the following 22 years of treatment with hemin. Since 2021, her porphyria has been managed with givosiran. Whilst receiving this subcutaneous therapy, the patient has not had any further attacks.

In addition to the treatment with hemin or givosiran, patients with acute intermittent porphyria should be counselled to avoid triggers, including alcoholic beverages, smoking, caloric restriction, intake of sex hormones and infections [8]. Drugs that are safe or unsafe need to be identified [20]. Patients should know that the best way to remain asymptomatic is by eliminating precipitants. This will minimize the risk of flares and optimize disease management.

The only cure for acute intermittent porphyria is orthotopic liver transplantation; however, this should be reserved for patients with severe recurrent acute attacks (defined as four or more attacks per year), highly impaired quality of life [21–23] and a significant risk of morbidity and mortality [24]. In rare cases, therefore, transplantation is a treatment option if all other therapeutic approaches fail. At Graz University Medical Center, none of our five patients with acute intermittent porphyria has received a liver transplantation so far. Transplantation of the liver of a patient with acute intermittent porphyria to a recipient with another cause of end-stage liver disease can result in the development of acute intermittent porphyria in the recipient [25, 26].

### Dr. G. J. Krejs:

Acute intermittent porphyria is an autosomal dominant disorder caused by partial deficiency in porphobilinogen deaminase (PBGD), also known as hydroxymethylbilane synthase (HMBS), the third enzyme in the heme biosynthesis pathway. In Europe, the genetic prevalence is 1 in 2000 [27]; however, the penetrance leading to clinical disease has been estimated to be less than 1% [28]. Women are more susceptible [26]. In addition to reduced PBGD activity, symptomatic acute intermittent porphyria requires a marked induction of the housekeeping isoform of the first enzyme in the heme biosynthetic pathway, ALAS1, which is upstream of PBGD in the heme synthesis pathway and the rate-limiting enzyme for heme synthesis in the liver. Marked ALAS1 induction leads to increased production and accumulation of the intermediates delta-aminolevulinic acid and porphobilinogen [27]. Patients with acute intermittent porphyria present with various clinical symptoms affecting the autonomic, central and peripheral nervous system. Manifest or overt acute intermittent porphyria is considered when patients develop typical acute neurovisceral attacks, neurologic symptoms such as peripheral neuropathy but also SIADH (hyponatremia) and psychiatric disturbances including hallucinations [6, 7, 29]. More than 80% of patients present with pseudoacute abdomen which usually lasts hours or days, does not respond well to analgesics and is often disproportional to the findings on physical examination [30]. Thus, patients often undergo exploratory laparotomy with a negative result. Henry Bockus, who organized the first World Congress of Gastroenterology in 1960, summarized causes simulating acute abdomen [31]. Besides acute intermittent porphyria, they include some infectious diseases such as typhoid fever, leptospirosis, malaria or hantavirus infection, and a variety of specific clinical states such as diabetic ketoacidosis due to possible irritation of the peritoneal pain receptors by osmotic mechanisms or by acid-base disturbance [32], sickle cell crisis with impaired capillary and subsequent organ perfusion caused by vascular occlusion [33], mucosal swelling in angioedema [34, 35], acute glaucoma which triggers a direct oculoabdominal reflex involving the trigeminal nerve and the vagal visceral motor and visceral sensory branches [36], chronic lead poisoning causing negative effects on intestinal motility [37], the intestinal neural web or smooth muscles mediated by lead or increased levels of delta-aminolevulinic acid [38, 39], and spontaneous bacterial peritonitis [40]. As in the discussed patient, abdominal complaints in acute intermittent porphyria further frequently include constipation and abdominal distention [6]. Clinical features associated with adverse effects of porphyrins on the central nervous system include seizures, SIADH and porphyria-induced posterior reversible encephalopathy syndrome (PRES) [6]. Mechanistically, neurotoxic [41, 42] but also vasoconstrictive [6, 43] effects of delta-aminolevulinic acid are discussed. Due to its structural similarity to gamma-aminobutyric acid (GABA), accumulation of delta-aminolevulinic acid is further assumed to impair normal GABA function in the central nervous system (CNS) [44, 45]. Besides CNS symptoms, peripheral neuropathy is also common in acute intermittent porphyria and includes muscle weakness, sensory neuropathy (paresthesia, hypoesthesia, numbness and neuropathic pain) and paresis that may progress to respiratory paralysis [46, 47]. Severe cases may even require intubation and mechanical ventilation [43]. Electrophysiologic examination has found primary axonal motor neuropathy in patients with acute porphyric neuropathy [48–50]. On autopsy, axonal motor fibers were significantly more affected than sensory fibers [51]. Since fast axonal transport depends on energy and heme is required for aerobic metabolism, it can be speculated that heme deficiency leads to axonal damage and subsequent peripheral neuropathy [6].

**Fig. 1:**
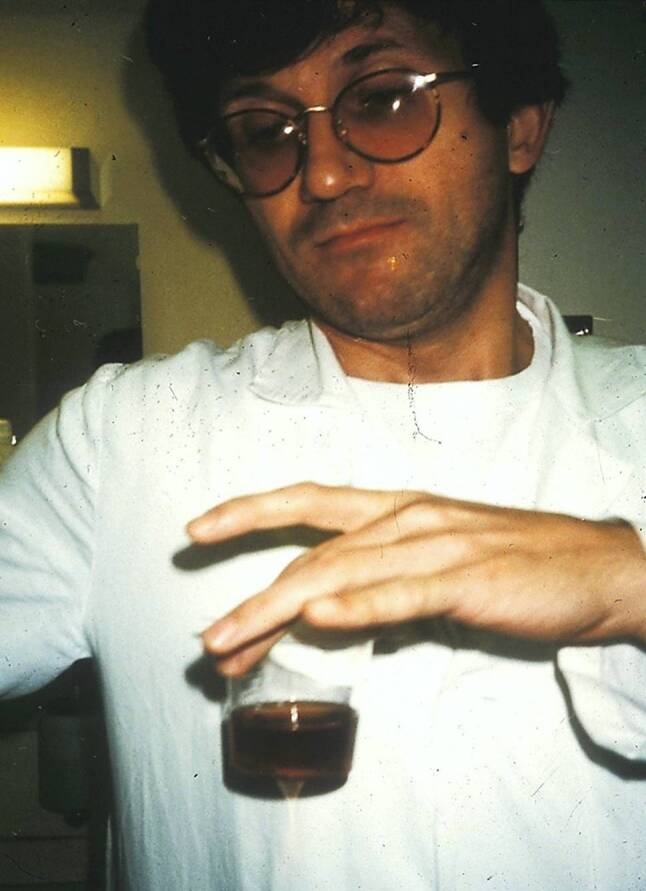
Resident Dr. A.J. Eherer showing characteristic burgundy red colored urine due to formation of porphobilin after 1 hr of exposure to light and room air when the diagnosis of acute intermittent porphyria was established in 1994 in the patient who is currently receiving givosiran as the first patient in Austria described by Dr. Stadlbauer (see text). (Copyright © privat)

Another hallmark indicating acute intermittent porphyria is a change in urine appearance. Exposure to light, oxygen or heat causes conversion of porphobilinogen to porphobilin and colors the urine burgundy red (Fig. 1). The suspected macrohematuria noted earlier in the discussed patient may reflect a former episode of acute intermittent porphyria since urinary test sticks have not been positive for erythrocytes at that time. During the current flare, the urinary test stick was falsely positive for bilirubin because of increased excretion of porphyrins. In general, urinary test sticks will never be positive for bilirubin at normal serum bilirubin levels and must always be questioned.

Acute intermittent porphyria is a metabolic disease with a wide variety of clinical manifestations. Although there is a high genetic prevalence, the overall genetic susceptibility factors underlying penetrance remain unknown [7]. Therefore, physicians should be aware of this diagnosis whenever a patient presents with pseudoacute abdomen, neurologic or psychiatric symptoms and hyponatremia.

After our patient was readmitted following surgery, the diagnosis of acute intermittent porphyria was made very promptly by the treating physicians. One possible association between hyponatremia and the patient’s history of colonic lavage comes to mind but can be discarded due to the electrolyte constellation discussed by Dr. Ribitsch with a high rather than low urinary sodium concentration in our patient. Colonic lavage, particularly for the cleansing prior to colonoscopy, resulting in hyponatremia has occasionally been reported in the last 25 years [52, 53]. The senior author even knows of one fatal case under such circumstances.

## Final diagnosis

Acute intermittent porphyria
